# Effects of thermal and oxygen conditions during development on cell size in the common rough woodlice *Porcellio scaber*

**DOI:** 10.1002/ece3.6683

**Published:** 2020-08-18

**Authors:** Andrzej Antoł, Anna Maria Labecka, Terézia Horváthová, Anna Sikorska, Natalia Szabla, Ulf Bauchinger, Jan Kozłowski, Marcin Czarnoleski

**Affiliations:** ^1^ Institute of Environmental Sciences Jagiellonian University Kraków Poland; ^2^ Institute of Soil Biology Biology Centre CAS České Budějovice Czech Republic; ^3^ Nencki Institute of Experimental Biology Polish Academy of Sciences Warsaw Poland

**Keywords:** life history evolution, metabolic rate, optimal cell size, temperature–size rule

## Abstract

During development, cells may adjust their size to balance between the tissue metabolic demand and the oxygen and resource supply: Small cells may effectively absorb oxygen and nutrients, but the relatively large area of the plasma membrane requires costly maintenance. Consequently, warm and hypoxic environments should favor ectotherms with small cells to meet increased metabolic demand by oxygen supply. To test these predictions, we compared cell size (hindgut epithelium, hepatopancreas B cells, ommatidia) in common rough woodlice (*Porcellio scaber*) that were developed under four developmental conditions designated by two temperatures (15 or 22°C) and two air O_2_ concentrations (10% or 22%). To test whether small‐cell woodlice cope better under increased metabolic demand, the CO_2_ production of each woodlouse was measured under cold, normoxic conditions and under warm, hypoxic conditions, and the magnitude of metabolic increase (MMI) was calculated. Cell sizes were highly intercorrelated, indicative of organism‐wide mechanisms of cell cycle control. Cell size differences among woodlice were largely linked with body size changes (larger cells in larger woodlice) and to a lesser degree with oxygen conditions (development of smaller cells under hypoxia), but not with temperature. Developmental conditions did not affect MMI, and contrary to predictions, large woodlice with large cells showed higher MMI than small woodlice with small cells. We also observed complex patterns of sexual difference in the size of hepatopancreatic cells and the size and number of ommatidia, which are indicative of sex differences in reproductive biology. We conclude that existing theories about the adaptiveness of cell size do not satisfactorily explain the patterns in cell size and metabolic performance observed here in *P. scaber*. Thus, future studies addressing physiological effects of cell size variance should simultaneously consider different organismal elements that can be involved in sustaining the metabolic demands of tissue, such as the characteristics of gas‐exchange organs and O_2_‐binding proteins.

## Graphical abstract

Cell size and metabolic rate were checked in common rough woodlice. Animals were reared in two different temperatures and two different oxygen conditions. Complex pattern in cell size was discovered with most profound changes attributed to body size and weak link between cell size and metabolic rate occured.

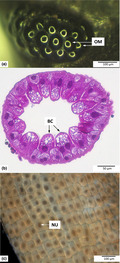

## 1 INTRODUCTION

Oxygen supply is typically considered a potential limiting factor for aquatic organisms (Bonvillain, Rutherford, & Kelso, 2015; Czarnoleski, Ejsmont‐Karabin, Angilletta, & Kozlowski, 2015; Hoefnagel & Verberk, 2015; Kiełbasa, Walczyńska, Fiałkowska, Pajdak‐Stós, & Kozłowski, 2014; Verberk et al., 2016; Walczyńska, Labecka, Sobczyk, Czarnoleski, & Kozłowski, 2015; Woods, 1999). Nevertheless, obtaining adequate amounts of oxygen may also be challenging for terrestrial organisms, especially those that live at high elevations or occupy habitats with high microbial respiration, periodic flooding, or high snow coverage (Hoback & Stanley, 2001; Paim & Beckel, 1964; Peacock, 1998), as well as if they simply undergo phases with much higher metabolic rates (Czarnoleski, Dragosz‐Kluska, & Angilletta, 2015). The need to supply oxygen to meet the metabolic demand of tissues has led to the selection of organisms with various adaptations, such as behavioral avoidance of warm microenvironments (Antoł, Rojek, Singh, Piekarski, & Czarnoleski, 2019; Hicks & Wood, 1985; Wood & Gonzales, 1996), the ability to access alternative oxygen sources (e.g., air oxygen for aquatic organisms; Scott et al., 2017) or the ability to switch to anaerobic metabolism (Wright & Ting, 2006). In this study, we considered that developmental plasticity in cell size may be another adaptive response aimed at satisfying metabolic oxygen needs. The adaptive value of cell size has long been overlooked despite accumulating evidence that it can co‐vary with body mass within species (Arendt, 2006; Czarnoleski, Cooper, Kierat, & Angilletta, 2013) and between species (Czarnoleski et al., 2018; Kozłowski, Czarnoleski, François‐Krassowska, Maciak, & Pis, 2010), change with genome size (Gregory, 2001) and cell ploidy (Hermaniuk, Rybacki, & Taylor, 2016, 2017), and respond to developmental conditions such as temperature (Arendt, 2006; Azevedo, French, & Partridge, 2002; Czarnoleski, Dragosz‐Kluska, et al., 2015; Czarnoleski, Ejsmont‐Karabin, et al., 2015; Czarnoleski, Labecka, & Kozlowski, 2015; French, Feast, & Partridge, 1998; Hermaniuk, Rybacki, & Taylor, 2016), oxygen supply (Heinrich, Farzin, Klok, & Harrison, 2011; Zhou et al., 2007), and food supply (Arendt, 2006).

The theoretical framework for our study, namely, the theory of optimal cell size (TOCS), integrates ideas regarding the adaptiveness of cell size developed by earlier works (Atkinson, Morley, & Hughes, 2006; Czarnoleski et al., 2013; Czarnoleski, Dragosz‐Kluska, et al., 2015; Czarnoleski, Ejsmont‐Karabin, et al., 2015; Czarnoleski, Labecka, et al., 2015; Czarnoleski et al., 2017, 2018; Davison, 1956; Hermaniuk et al., 2016; Kozłowski, Konarzewski, & Czarnoleski, 2020; Kozłowski, Konarzewski, & Gawelczyk, 2003; Maciak, Bonda‐Ostaszewska, Czarnoleski, Konarzewski, & Kozlowski, 2014; Szarski, 1983; Walczyńska et al., 2015; Woods, 1999). In line with Szarski (1983), Kozłowski et al. (2003), and Kozłowski et al. (2020), TOCS considers that organismal strategies span a frugal‐wasteful continuum that is largely dictated by cell size, with a frugal strategy (large cells in tissue) having low metabolic requirements at the costs of poor capacity to maintain high activity and rapid development compared with a wasteful strategy (small cells in tissue). In understanding the link between cell size and physiology, it should be considered that a plasma membrane (also called cell membrane or plasmalemma) is an important transport hub that affects the physiological capacity of cells. However, the operational state of a plasma membrane requires building and maintaining ionic gradients on the cell surface and turning over phospholipids and membrane proteins, and such maintenance requires substantial amounts of ATP and resources (Engl & Attwell, 2014; Rolfe & Brown, 1997). Consequently, TOCS predicts that frugal strategies would connect with organisms characterized by larger cells, which would reduce the amount of plasma membrane per tissue/organ volume and, thus, the physiological load caused by plasma membrane maintenance. In contrast, wasteful strategies would involve organs and tissue built of smaller cells. On the other hand, TOCS considers that organs built of smaller cells with more plasma membranes may have an increased capacity to deliver oxygen through tissue to mitochondria. This is because oxygen diffuses better in lipids than in water (Subczyński, Hyde, & Kusumi, 1989), and thus, as suggested by the results of Subczyński and Hyde (1998), plasma membranes in tissue may serve as oxygen transportation pathways. Ultimately, the theoretical framework of TOCS considers that the fitness cost and benefit of a heavily wasteful versus frugal strategy depend on the selective pressure of the environment, especially the balance between metabolic demand and oxygen supply. In particular, wasteful strategies with smaller cells would be adaptive in warm and hypoxic conditions, whereas frugal strategies with large cells would benefit organisms in cold and normoxic conditions.

To address the predictions of TOCS, we studied the effects of thermal and oxygen conditions during development on cell size in the common rough woodlice (*Porcellio scaber*), a species of terrestrial isopods. Typically, terrestrial isopods inhabit litter environments with intense decomposition and thus are likely to experience hypoxic conditions in their natural habitats (Wright & Ting, 2006). Having evolved various land adaptations, such as pleopodal lungs, water conducting systems, or conglobating behavior (Cloudsley‐Thompson, 1988; Hornung, 2011), terrestrial isopods are sometimes regarded as the best land adapted order of crustaceans (Hornung, 2011). Previous studies of terrestrial isopods showed that oxygen deficiency in the air affects their mobility, thermal performance and thermal preferences (Antoł et al., 2019), respiration rate and hemolymph lactate level (Wright & Ting, 2006), lung size (Antoł et al., 2020), and the length of aquatic/air phases during marsupial development of offspring (Horváthová, Antoł, Czarnoleski, Kozłowski, & Bauchinger, 2017). In this study, we assessed the cell size of woodlice in the eye, hepatopancreas and hindgut and evaluated how it changes with developmental conditions. To the best of our knowledge, no earlier study has focused on the cell size of isopods, especially considering the plastic developmental responses of cells to environmental conditions. Importantly, by considering different cell types with distinct physiological functions, we were able to address whether cell size in a given tissue should be entirely viewed with reference to the specific functions of a tissue or whether it undergoes (at least to some degree) organism‐wide coordination in different tissue types. Interestingly, most earlier studies concerning cell size variance focused on single cell types, assuming that cells undergo coordinated growth and proliferation in different tissues (Heinrich et al., 2011; Starostová, Konarzewski, Kozłowski, & Kratochvíl, 2013). However, emerging evidence shows that while such control may indeed occur, organs and tissues can group according to the size responsiveness of their cells, indicating the need to consider tissue‐specific cell functions (Czarnoleski, Ejsmont‐Karabin, et al., 2015; Czarnoleski et al., 2017, 2018; Kozłowski et al., 2010). Following TOCS, we predicted that the cell size of woodlice would generally decrease in response to either elevated metabolic demands (warm) or lowered oxygen supply (hypoxia), and we expected that this effect would be more pronounced under combined warm and hypoxic conditions (hypothesis 1). Before we sampled tissues for the determination of cell size, we assessed the capacity of each animal to meet increased metabolic demand under poor oxygen supply. For this purpose, we measured the magnitude of a metabolic increase, following a transfer of each woodlouse from cool and normoxic conditions to warm and hypoxic conditions. Following TOCS, we predicted that small‐cell woodlice would better tolerate such a challenge, which would be manifested by a higher increase in metabolic rate than large‐cell conspecifics (hypothesis 2). Importantly, although one could expect cell size differences among different cell types, addressing this possibility among the studied tissues was beyond the scope of our study, since differences in cell physiology among tissues could be largely attributed to function‐related properties of cells, such as their shape (neurons vs. epithelial cells) or organellular content.

## 2 MATERIALS AND METHODS

### 2.1 Animals

This work was part of a long‐term study of *P. scaber*, and its methods are already available elsewhere (e.g., Antoł et al., 2020; Horváthová et al., 2017; Horváthová, Antol, et al., 2015). Briefly, adults of *P. scaber* were collected in autumn (2013) and spring (2014) in an old backyard in Kraków, Poland (50°04ʹ15.9″N 19°56ʹ21.9″E). After sexing, females and males were transferred to separate plastic boxes with moist sand and broken clay pots as shelters, and the boxes housing the animals were kept in a thermal cabinet (Pol‐Eko Aparatura, Wodzisław Śląski, Poland) set to 15°C during the day and to 8°C at night with a 12L:12D photoperiod. Note that these conditions mimicked autumn/spring conditions in the source population. Animals were fed ad libitum with a mixture of dry black alder (*Alnus glutinosa*) and European ash (*Fraxinus excelsior*) leaves from a nearby forest. Note that the field‐collected woodlice served as the parental generation for the new laboratory‐created generation of woodlice, which was subjected to our developmental experiment.

### 2.2 Developmental experiment

The experiment aimed to allow woodlice to develop in one of four environments corresponding to each combination of two temperatures (15 and 22°C) and two oxygen concentrations in the air (10% and 22%). The environments were generated in two thermal cabinets (Pol‐Eko Aparatura) set to either 15 or 22°C, with the help of four Plexiglass chambers (40 × 50 × 55 cm), with two chambers placed in each cabinet. One of the two chambers per cabinet was set to normoxia (22%), and the other was set to hypoxia (10%). The oxygen conditions inside the chambers were controlled by a four‐channel gas regulator ROXY‐4 (Sable Systems International (SSI), Las Vegas, NV, USA). The regulator was connected to nitrogen and oxygen gas tanks (Air Products Sp. z o.o., Kraków, Poland) to supply the normoxic chambers with oxygen or the hypoxic chambers with nitrogen in the amounts necessary to create the experimental oxygen levels. Note here that we set the normoxic conditions to 22% O_2,_ which was slightly above the oxygen concentration in the atmospheric air. As the atmospheric air was slowly leaking into the normoxic chamber, the system was immediately pumping in oxygen until the required level of 22% O_2_ was achieved. Inside the chambers, air circulation was maintained by fans, and the relative humidity was controlled by four dew point generators DG‐4 (SSI) set to 75% relative humidity and connected to the chambers via PP2 pumps (SSI). The temperature and humidity inside the chambers were monitored with Hygrochron iButtons (Maxim/Dallas Semiconductor, San Jose, CA, USA) placed within the food leaves and shelters of the woodlice. Note that the relative humidity experienced by the experimental animals reached 98%, which closely resembles the microhabitat humidity of our source population (Horváthová, Antol, et al., 2015).

To obtain a new generation for the experiment, the field‐collected parental woodlice were randomly allocated for mating in 28 boxes, with 40 males and 50 females per box. The mating groups were randomly allocated to one of the four experimental environments to ensure that all animals from the new generation had already been exposed to the treatment conditions at the egg stage. According to McQueen and Steel (1980), the mating of parental animals was synchronized and stimulated by a sudden switch to a long‐day photoperiod (16L:8D). The mating groups were checked weekly for gravid females (eggs or juveniles in a brood pouch – marsupium). Gravid females were immediately placed in isolation in 100 ml plastic boxes (the habitat inside the boxes was created as described in the *Animals* section). Every week, the boxes with gravid females were sprayed with water, and leaves were added. If free living stages of offspring were observed, the female parent was removed from the box. The offspring were fed dry leaves every week (see *Animals* section). To ensure access to gut microsymbionts, two‐week‐old juveniles were provided with a body of a dead adult conspecifics (Horváthová, Kozłowski, & Bauchinger, 2015). From the 4th week of postmarsupial life, the offspring were supplemented with one dried mealworm per box per week (*Tenebrio molitor*). At the age of 20 weeks, we pooled experimental animals of the same sex from all boxes and formed single‐sex groups (20 animals per group). Each group was placed in a box where the animals remained until the end of the experiment. When the females reached a body mass indicative of mature woodlice in our source population in the field (76.83 mg on average, Antoł & Czarnoleski, 2018), we added several males to each box with the females to provide conditions for physiological maturation (Kight, 2008). When the animals reached the age of 18 months at 22°C or 28 months at 15°C, we terminated the experiment. For the animals that survived to this stage, we conducted measurements of respiration rate under two conditions and then performed dissections for cell size measurements.

Initially, all four experimental treatments involved a new generation of woodlice produced by the parental animals collected in the field in autumn 2013. Unfortunately, due to failure of one of our thermal cabinets, we lost animals from the warm treatment. Given the expected developmental lag between cold and warm woodlice, we decided to continue the cold treatment and run the warm treatment again a few months later using descendants of the parental animals collected in spring 2014. Both samples of parental animals (autumn 2013 and spring 2014) originated from the same source population and were acclimated to the same laboratory conditions prior to egg laying, including mating stimulation via the transition through the winter/spring phases. Therefore, we have good reason to believe that the parental animals collected in the autumn and the spring gave birth to phenotypically similar offspring, not biasing our comparisons of animals developed in the laboratory in cold and warm treatments.

### 2.3 Magnitude of metabolic increase (MMI)

Respiration measurements were performed on the woodlice available at the end of our experiment (age: 18 months at 22°C and 28 months at 15°C). Importantly, after this step, we utilized the same individual woodlice in histological analyses to assess cell size in the three evaluated tissues (see later Methods).

Our respiration measurements aimed to test whether animals from different treatments (and, possibly, with different cell sizes) cope differently with metabolically challenging conditions. For this purpose, we measured the respiration rate of each animal twice: first in less demanding cold/normoxia conditions (15°C combined with 22% O_2_) and then in more demanding warm/hypoxia conditions (22°C combined with 10% O_2_). Ultimately, each animal was characterized by the magnitude of metabolic rate increase (MMI), calculated as the ratio of the latter to former respiration rate. Before and after respiration measurements, animals were maintained in their original experimental treatments, and they were kept in 100 ml plastic boxes with a leaf of European ash and black alder as food sources, a piece of wet paper as a water source and a piece of a broken clay pot as a shelter. Prior to respirometry measurements in either cold/normoxia or warm/hypoxia, animals were acclimated to each condition for two days.

For respiration measurements, we used an eight‐channel MUX multiplexer (SSI) with 7 channels connected to respiratory chambers dedicated for animals and one empty channel for a baseline. We used glass tubes (length: 7 cm, diameter: 2 cm) as respiratory chambers. To limit the locomotor activity of animals, we decreased the volume of each respiratory chamber by placing a smaller plastic tube filled with artificial cotton wool inside the chambers. The respiration chambers were placed in a thermal cabinet for temperature control during the measurements. We obtained the respiration rate by measuring CO_2_ production in a flow‐through system (SSI). We used the gas stream either from the outside (in case of normoxia measurements) or from a tank with a 10% O_2_:90% N_2_ gas mixture (Gaz Centrum, Kraków, Poland) for measurements in hypoxia. Before entering the system, the inflowing gas was scrubbed for H_2_O with the use of calcium sulfate (Drierte Co. Ltd, Xenia, USA) and for CO_2_ with the use of soda lime (Drierte Co. Ltd). To pump the gases, we used an SS4 Subsampler (SSI). The flow rate was set to 40 ml/min with a mass flow controller (Sidetrack Mass Flow Controller, Sierra Instruments, Monterey, CA, OH, USA). The relative humidity inside the respiration chambers was set to 85% (regulated with a DG‐4 Dewpoint Generator, SSI, and controlled with an RH‐300, SSI). The air leaving the experimental chamber was dried with magnesium perchlorate (Merck KGaA, Darmstadt, Germany). Every second, an infrared CO_2_ gas analyser (Li‐7000, Li‐Cor, Lincoln, NE, USA) recorded the $\dot{V}$ CO_2_ (in ppm) in the air leaving the experimental chamber. For each animal, respiration data were recorded for 10 consecutive minutes, after which data from the baseline were recorded for 5 min. The recorded $\dot{V}$ CO_2_ was converted to ml CO_2_ min^−1^, baseline‐ and drift‐corrected with ExpeData software (SSI). Ultimately, for each animal, we calculated the mean CO_2_ production during a 2.5‐min time interval when the mean rate of respiration reached its lowest value, first under cold/normoxia and then under warm/hypoxia, and these measurements were used to calculate MMI.

### 2.4 Cell size

With the goal of collecting cell size information from as many different tissues and organs as possible, we assessed cell size for three different cell types: cells forming ommatidia in the eye, B cells in the hepatopancreas, and epithelial cells in the hindgut. These three organs originate from two germ layers, ectoderm (hindgut and ommatidia) and endoderm (hepatopancreas; Hames & Hopkin, 1989). This approach allowed us to make generalizations regarding cellular composition in other tissue types, but the simultaneous consideration of even more cell types would certainly increase the generality of our findings significantly. Importantly, each of the studied cell types performs different and highly specialized physiological and organismal functions. In isopods, each ommatidium is formed by a constant number of ten cells (Nemanic, 1975), which allowed us to treat the size of an ommatidium facet as a proxy of cell size. Interestingly, cells forming the compound eye of isopods can flexibly change their organellular content according to light conditions (Nemanic, 1975). Hepatopancreatic B cells are large and have a pear shape, and they project apically into the lumen of the organ (Hames & Hopkin, 1991). The hepatopancreas of isopods was reported to take part in enzyme secretion, nutrient absorption, and heavy metal handling (Žnidaršič, Štrus, & Drobne, 2003), and it is involved in interactions with symbiotic microorganisms of isopods (Wang, Brune, & Zimmer, 2007). Hindgut epithelial cells form one‐layered epithelium of the hindgut and are involved in the processing of undigested food as well as fluid recycling through typhlosole channels (Hames & Hopkin, 1989).

To measure cell size in the studied woodlice, the animals used in respirometry measurements were weighed to the nearest 0.001 mg on a microbalance and then dissected to obtain the head, hepatopancreas, and hindgut. Animals were decapitated with a scalpel in a Petri dish. The remaining body was submerged in 1× PBS (Avantor Performance Materials, Gliwice, Poland), and the hindgut and hepatopancreas were extracted from the body. Food residuals were washed out from the hindgut with 1× PBS, after which both organs were fixed in 10% buffered formalin (Chempur, Piekary Śląskie, Poland).

**FIGURE 1:**
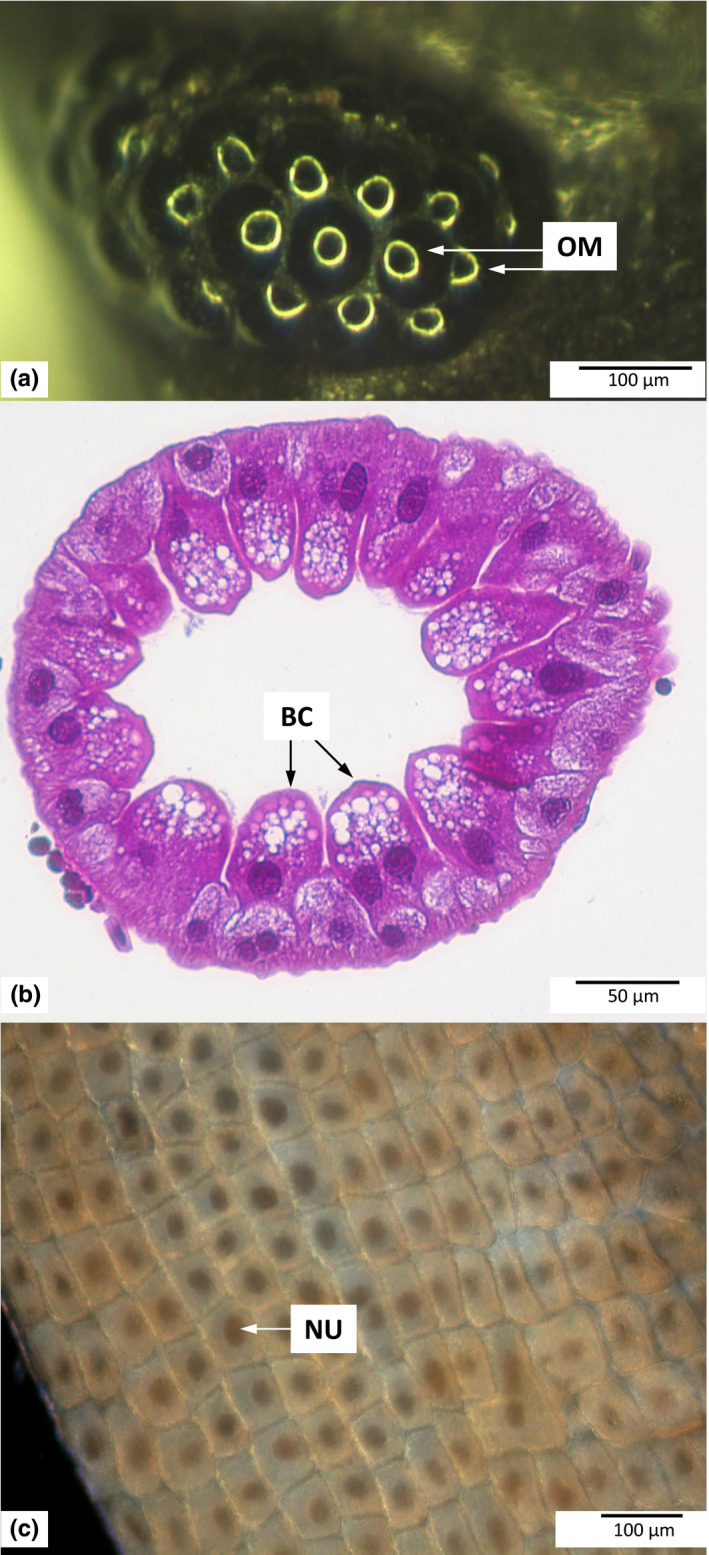
Examples of organs of *Porcellio scaber* that were used to assess cell size in individuals reared in two different oxygen levels (10% and 22%) and temperatures (15 and 22°C): (a) eye (top view); (b): hepatopancreas; (c): hindgut epithelium. Abbreviations: BC—B cells; NU—epithelial cell nucleus; OM—ommatidia. The ring visible in the ommatidium image is a reflection of light and serves as a tool for orienting a particular ommatidium perpendicularly with respect to the objective

Each freshly cut head was used to image ommatidia in the eyes. Additionally, the total number of ommatidia in the eyes was counted to explore whether changes in the size of ommatidia correspond to changes in the number of ommatidia in the compound eye. We imaged ommatidia in both eyes under 63× magnification with a uEye digital camera (IDS Imaging Development Systems GmbH, Obersulm, Germany) and a stereoscopic microscope SZY 10 (Olympus, Tokyo, Japan). The heads were impaled on a pin mounted in plasticine and lit with ring light (KL‐RL‐9/1000‐3, Olympus). First, we took overview images of the entire eye and the frontal and back parts of the eye. Then, the head was positioned to obtain a perpendicular orientation of the singular ommatidium to the camera. The perpendicularity was controlled with the central settlement of the position of the light reflected by the ommatidium facet (Figure 1a). Only ommatidia that were not along the border of the eye were imaged to avoid possible shape irregularities caused by contact with the cephalic shield. The ommatidia measurements were performed in ImageJ software (NIH, Bethesda, USA). We split each image into separate color channels and performed measurements on the green channel to ensure the best visibility of ommatidia borders. After contrast enhancement, we fitted an ellipse to each ommatidium and measured its area to the nearest 0.001 μm^2^.

The fixed hepatopancreas was washed three times for 30 min in 1× PBS. Next, the organ was dehydrated in a graded series of ethanol (70%, 80%, 90%, 96%, and 99.8%), cleared in ST Ultra (Leica, Wetzlar, Germany) and embedded in Paraplast Plus (Leica). Serial cross‐sections (4‐µm thick) were cut with a rotary microtome (Hyrax M55, Zeiss, Oberkochen, Germany). Histological slides were deparaffinized, rehydrated, and stained with Ehrlich hematoxylin (Carl Roth, Karlsruhe, Germany) for ten minutes and then with a 1% ethanol solution of eosin Y (Analab, Warszawa, Poland) for five minutes. Then, slides were dehydrated in 96% ethanol and a mixture of phenol (Avantor Performance Materials) and xylene (Avantor Performance Materials), cleared in xylene (Avantor Performance Materials), and embedded in DPX (Aqua‐Med Zpam‐Kolasa sp.j., Łódź, Poland). We took images of hepatopancreas cross‐sections in ZEN image acquisition software (Zeiss) under a light microscope (Eclipse 80i, Nikon, Tokyo, Japan) in a bright field using 200× microscope magnification. Using ImageJ software, we measured B cells (Hames & Hopkin, 1991) with visible nuclei and nucleoli (Figure 1b). We outlined cells along the plasma membrane to measure the cell area to the nearest 0.001 μm^2^. Then, we calculated the mean cell size per individual, and this value was used in the analysis.

The hindgut after fixation was washed two times for five minutes in 1× PBS and then stained in an Eppendorf‐like tube for 30 min with Gill II hematoxylin (Carl Roth). Organs were washed in tap water, cut and placed in one drop of tap water on the surface of a glass slide before being covered such that the luminal side of the hindgut epithelium was oriented toward the cover slide. We captured images of the epithelium under a light microscope (Eclipse 80i) in a dark field using 100× microscope magnification (Figure 1c) and measured the epithelial cell size according to the method proposed by Czarnoleski et al. (2017) for hepatocytes using ImageJ software. In each image, we outlined a circular area covering the largest area of the tissue with cells with visible nuclei. To calculate the mean size of the hindgut epithelial cells for each individual, we measured the area of all circles from that individual and divided the area by the total number of cells inside. Cells located on the border were included if the majority of their surface was inside the circle. If such classification was ambiguous, the cell was randomly classified at a rate of 50% as being located inside the area. Then, we counted the total number of cells in all circles per individual, and the mean cell area measured to the nearest 0.001 μm^2^ per individual by dividing the total area of measured circles per individual by the total number of cells for that individual.

In total, we measured 4–22 ommatidia (average 14.2), 3–95 hepatopancreas cells (average 35.7), and 46–273.5 (average 136.4) hindgut epithelium cells per individual woodlouse. Note that these ranges mainly reflect a change in the number of cells available for measurements with woodlouse body size.

### 2.5 Statistical analysis

We collected a full dataset from 111 animals (61 males and 50 females), which included information on sex, body mass, MMI, and cell size measurements for each of the three cell types. Statistical analysis and graphic presentation of the results were conducted in R software (R Core Team, 2018) with lme4 (Bates, Mächler, Bolker, & Walker, 2015), lmerTest (Kuznetsova, 2017), effects (Fox & Weisberg, 2018), and ggplot2 (Wickham, 2016) packages. Prior to the analysis, data on cell size were normalized and then analyzed with principal component analysis (PCA). This analysis was performed to (a) integrate information about cell size in different organs and (b) evaluate cell size correlations among different tissues. In the subsequent analyses, we used scores of extracted principal components (PC) as indices of integrated information on cell sizes. Following Quinn and Keough, (2002), we considered that the variance explained by PCs with eigenvalues >1 should have much higher biological relevance than the variance explained by PCs with eigenvalues <1. Consequently, testing our a priori hypotheses (1 and 2), we focused primarily on PCs with eigenvalues >1.

To test hypothesis 1 (metabolic demand and oxygen supply during development induce developmental changes in cell size) and hypothesis 2 (MMI is higher in woodlice with smaller cells), we analyzed our cell size measures (PC scores) and MMI (as dependent variables) with the help of two separate general linear models (GLMs) that had the same structure: rearing temperature and oxygen conditions as two fixed factors, sex as another fixed factor, and body mass of woodlice as a numeric covariate. The models also included an oxygen × temperature interaction, which allowed us to explore whether the effects of increased metabolic demand (warm) are magnified by low oxygen supply (hypoxia); no other interactions were present in the model. The MMI data were log‐transformed prior to the analysis to meet the assumption of normality. Another GLM with a similar structure was used to analyze ommatidia number. If any of our GLM models revealed a significant interaction, we further explored differences between groups with Tukey's HSD test.

## 3 RESULTS

Our measures of cell size in different organs are presented in Table 1. Following our PCA of the cell size data (Table 2), we considered two principal components, PC1 and PC2, though only PC1 had a large enough eigenvalue (1.86) to regard it as significant (Quinn & Keough, 2002). Note that although we further analyzed the results of both PCs, our hypothesis testing involved the results of PC1, whereas the results of PC2 were analyzed for explorative purposes.

**TABLE 1:** Measurements of cell size (in μm^2^) for three organs (eye, hepatopancreas, and hindgut) of the common rough woodlice (*Porcellio scaber*) reared in two different temperatures (15 and 22°C) and two different oxygen levels (10% and 22% O_2_)

	Pooled data				Mean values per group							
					22°C				15°C			
					22% O_2_		10% O_2_		22% O_2_		10% O_2_	
	Mean value	Standard deviation	Minimal value	Maximal value	Female	Male	Female	Male	Female	Male	Female	Male
Eye (ommatidium)	5,811.688	793.7596	3,923.979	7,628.818	5,780.975	6,355.189	5,747.706	5,505.806	5,873.252	5,471.575	5,408.276	6,183.801
Hepatopancreas (B cells)	1,751.319	651.2294	446.3511	3,635.098	1,771.721	1,652.154	2,080.911	1,518.326	1,998.506	1,825.133	1,678.929	1,560.737
Hindgut (epithelial cell)	4,436.700	985.1412	1834.267	7,170.934	4,562.719	5,003.124	4,501.957	4,086.355	5,096.133	4,071.786	3,837.817	4,236.904

**TABLE 2:** Loadings in a principal component analysis (PCA) of cell size for three different cell types from *Porcellio scaber* woodlice reared at two temperatures (15 and 22°C) and two oxygen levels (10% and 22% O_2_)

Tissue	PC 1	PC 2
Ommatidia	0.56	−0.64
Hepatopancreas	0.51	0.77
Hindgut epithelium	0.65	−0.06
Eigenvalue	1.86	0.77
Explained variance	62%	26%

Note

PC1 and PC2 scores were used as integrated measures of cell size for hypothesis testing (Table 2).

The results of PC1 showed that cell size in the hepatopancreas and the hindgut and ommatidia size were positively loaded on PC1; therefore, higher scores indicate larger cells in these three cell types. The results of PC2 showed that the size of hepatopancreas cells and ommatidia was also partially loaded on PC2, but in opposite directions, higher scores were associated with larger hepatopancreatic cells and smaller ommatidia. The hindgut epithelium cell size did not contribute significantly to PC2. Note that the simultaneous contributions of cells in the hepatopancreas and ommatidia to PC1 and PC2 indicate that the variance in the size of these three cell types can be divided into two independent parts (though these parts are unequal because PC1 explained a much larger part of the variance than PC2), which have two different natures reflected in the structure of the two PCs.

**FIGURE 2:**
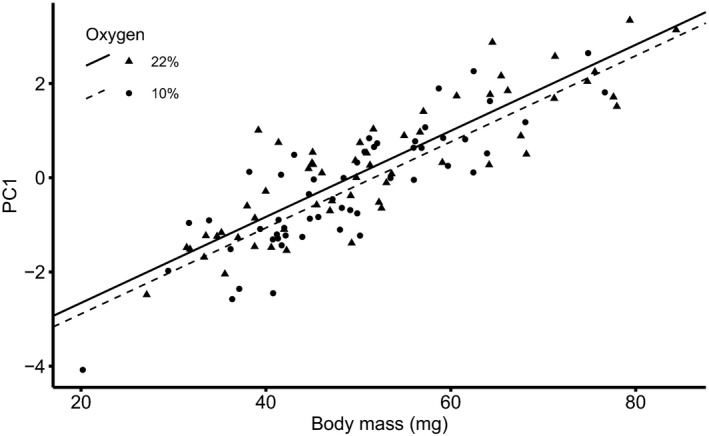
The first principal component (PC1) was positively correlated with ommatidia, hepatopancreas cells and hindgut epithelial cells in *Porcellio scaber* (see the loadings in Table 1), and its values increased with increasing body mass. There was a tendency (*p* = .07) of PC1 to reach higher values in animals developed under 22% O_2_

The results of the GLM for PC1 scores (Table 3) showed that the scores increased with woodlouse body mass (*p* < .001; Figure 2). This means that larger woodlice were characterized by larger cells in ommatidia, hepatopancreas, and hindgut epithelium. There was a tendency of PC1 scores to have lower values in hypoxia than in normoxia, but it was not statistically significant (*p* = .07). This result indicates a trend toward smaller cells in ommatidia, hepatopancreas, and hindgut epithelium in hypoxic woodlice than in normoxic woodlice. The effects of sex, temperature, and the oxygen × temperature interaction were not significant.

**TABLE 3:** Results of four general linear models (GLM) for cell size measures (PC1 and PC2), the magnitude of metabolic increase (MMI) and ommatidia number in *Porcellio scaber* reared at different temperatures (15 and 22°C) and oxygen levels (10% and 22%)

Factor	*df*	PC1		PC2		logMMI		Ommatidia number	
		*F*	*p*	*F*	*p*	*F*	*p*	*F*	*p*
Sex	1	0.71	.40	7.95	**.006**	0.17	.68	2.94	.09
Body mass	1	295.78	**<.001**	2.28	.13	4.62	**.03**	6.62	**.01**
Oxygen	1	3.37	.07	4.42	.04	1.03	.31	3.69	.06
Temperature	1	0.90	.35	1.57	.12	0.11	.75	30.46	**<.001**
Oxygen × temperature	1	0.74	.39	2.91	**.004**	1.79	.18	0.33	.57
Error	105								

Note

PC1 and PC2 were obtained via principal component analysis, and they represent integrated measures of cell size in ommatidia, the hepatopancreas, and the hindgut epithelium (see Table 2). The magnitude of metabolic rate increase (MMI) was calculated for each individual woodlouse upon transfer from less to more demanding conditions by dividing the CO_2_ production rate measured under more demanding conditions (22°C and 10% O_2_) by the CO_2_ production rate measured under less demanding conditions (15°C and 22% O_2_).

**FIGURE 3:**
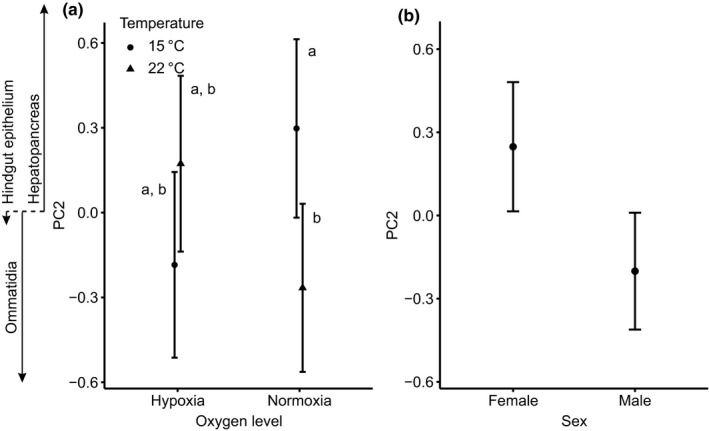
The second principal component (PC2) was positively correlated with the size of hepatopancreas cells and negatively correlated with ommatidia size in *Porcellio scaber* reared at two different oxygen levels (10% and 22%) and two different temperatures (15 and 22°C). (a) The values of PC2 differed significantly between animals reared at the two temperatures under normoxia (significant differences are marked with letters) and (b): between sexes. On the left side of the graph, the arrows depict loading values of different cell types. The correlation coefficients correspond to the values on the *Y* axis

The results of GLM for PC2 scores (Table 3) showed that males had lower scores than females (*p* = .006, Figure 3b). This means that compared with females, males were characterized by smaller hepatopancreatic cells and simultaneously larger ommatidia. Temperature and oxygen had an interactive effect on PC2 scores (*p* = .004, Figure 3a). The highest PC2 scores were achieved by animals that underwent development in either 22°C combined with 10% O_2_ or in 15°C combined with 22% O_2_. These conditions resulted in small ommatidia and large cells in the hepatopancreas (Figure 3a). Following our pairwise post hoc comparison of groups (Tukey's HSD test), the difference closest to the significance threshold was observed between animals reared at 22°C combined with 22% O_2_ and animals reared at 15°C combined with 22% O_2_ (*p* = .058). The body mass effect was insignificant (*p* = .13).

**FIGURE 4:**
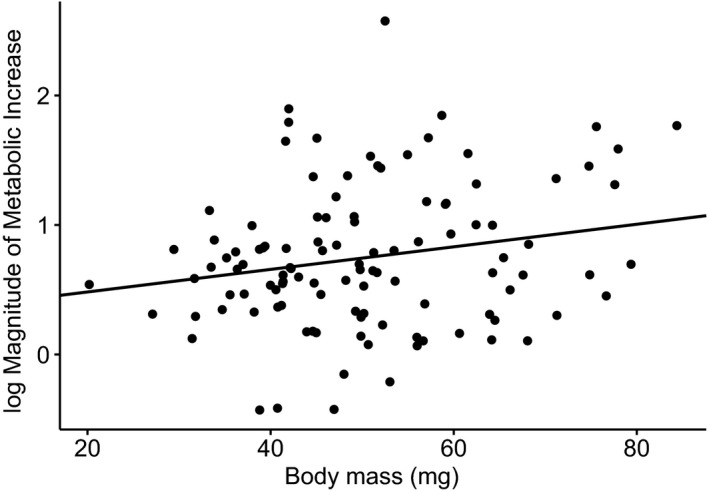
In *Porcellio scaber*, a transfer from 15°C and 22% O_2_ (less demanding condition) to 22°C and 10% O_2_ (more demanding condition) resulted in an increase in metabolic rate, but the increase was disproportionally higher among large woodlice than among small woodlice. The magnitude of metabolic increase (MMI) was calculated for each individual woodlouse by dividing the CO_2_ production rate under more demanding conditions by the CO_2_ production rate under less demanding conditions. The woodlice were reared under different developmental conditions comprising two oxygen levels (10% and 22%) and two temperatures (15 and 22°C)

The results of the GLM for the MMI (Table 3, Figure 4) showed that upon transition from cold, normoxic conditions to warm, hypoxic conditions, MMI did not differ between sexes and did not depend on the rearing conditions of the woodlice. However, the model showed an effect of woodlouse body mass on the MMI (*p* = .03), indicating that upon transition to more metabolically demanding conditions, large woodlice responded with a stronger increase in metabolic rate compared to small woodlice.

**FIGURE 5:**
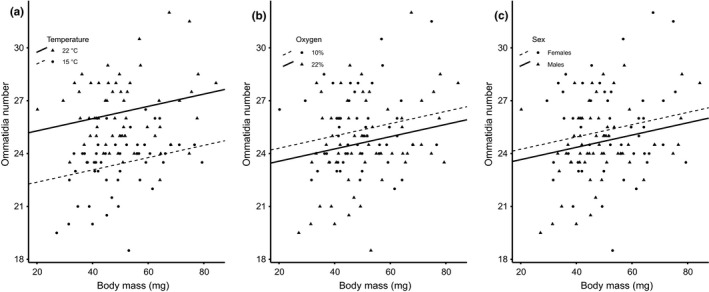
In *Porcellio scaber*, the number of ommatidia increased with body size. (a) Animals developed at high temperature (22°C) had more ommatidia than individuals developed at low temperature (15°C), and (b) animals developed at normoxia (10% O_2_) tended to have more ommatidia than animals developed at hypoxia (22% O_2_). (c) Females tended to have more ommatidia than males

The results of the GLM for ommatidia number (Table 3, Figure 5) showed that the number of ommatidia in the eye increased with woodlouse body mass (*p* = .01), animals reared in warm conditions had more ommatidia than animals reared in cold conditions (*p* < .001), females tended to have more ommatidia than males (*p* = .09), and animals reared in hypoxia tended to have more ommatidia than animals reared in normoxia (*p* = .06). We stress here that the two latter effects were weak and should be treated with caution. The effect of the oxygen × temperature interaction was not significant (*p* = .57).

## 4 DISCUSSION

### 4.1 Coordinated cell growth in tissues

A multicellular body consists of cells characterized by tissue‐specific properties, including cell size, cell shape, or organellular content, and there is no doubt that these differences are attributable to specialized functions performed by cells in different tissue and organs (Ginzberg, Kafri, & Kirschner, 2015). Although we should not expect that one universal size aids in different cell types meeting the requirements of tissue‐specific functions, our results call attention to intercorrelated changes in cell size in different tissues. By integrating information on cell size in the eye, hepatopancreas and hindgut of *P. scaber* with our PCA, we observed that the majority of cell size differences among individuals arose in a coordinated manner in different tissues (see our PC1, Table 1, Figure 2). Although we only studied three cell types, this finding strongly suggests that cell division and growth may be coordinated throughout the entire body rather than occurring totally independently in individual organs. The interlink between cell sizes in different organs/tissues has rarely been studied, but available evidence has revealed this phenomenon in different species, such as *Cornu aspersum* snails (Czarnoleski, Labecka, et al., 2015) and *Paroedura picta* geckos (Czarnoleski et al., 2017). Furthermore, interspecific comparisons of Hawaiian flies (Stevenson, Hill, & Bryant, 1995), amphibians (Kozłowski et al., 2010), birds (Czarnoleski et al., 2018; Kozłowski et al., 2010), mammals (Czarnoleski et al., 2018), and plants (Brodribb, Jordan, & Carpenter, 2013) as well as the results of experimental evolution in mice (Maciak et al., 2014) suggest that the size of cells in different organs can undergo highly coordinated evolution. Altogether, this emerging evidence highlights the importance of understanding the mechanisms of cell size control and organism‐wide coordination. These two phenomena are still not well recognized, but accumulating evidence has indicated that they might operate via evolutionarily conserved cellular signaling that targets biochemical pathways involved in cell size control, such as the target of rapamycin (TOR) and insulin regulatory pathway (De Virgilio & Loewith, 2006; Grewal, 2009), or via alterations in genome size (Gregory, 2001).

### 4.2 Cell growth contributes to an increase in body size

As the largest portion of the variance in cell size was attributed to PC1, cell size differences among individual woodlice could be largely attributed to differences in body size and to some extent to oxygen conditions during development. These results show that organs (at least those evaluated in this study) of large woodlice consisted of larger cells than organs of small woodlice, which resembles patterns revealed previously, for example, in tadpoles of *Spea hammondi* toad (Arendt, 2006), *D. melanogaster* flies (Czarnoleski et al., 2013), or *Lecane inermis* rotifers (Walczyńska et al., 2015). Interestingly, covariance between cell size and body size was also observed at the interspecific level, which strongly suggests that body size and cell size can undergo concerted evolution during species divergence (Czarnoleski et al., 2018; Kozłowski et al., 2010). Certainly, a change in body size can occur not only via cell size changes but also via changes in cell number, which was previously demonstrated in western spadefoot toads (Arendt, 2006) and fruit flies (De Moed, De Jong, & Scharloo, 1997). In *Drosophila* flies, for example, alterations in cell number accounted for 66% of the variance in body size (Böhni et al., 1999; Minelli, Maruzzo, & Fusco, 2010). For *P. scaber* studied here, we found that the number of ommatidia (eye elements built in isopods from a constant number of cells) increased in the eyes of larger woodlice, indicating that changes in cell number together with cell size contributed to changes in the size of eyes and possibly also in the body size of woodlice. Changes in cell size and cell number can also be viewed as components of ecological patterns in body size, which was envisioned to reflect adaptive shifts in life history, combined with tuning of cellular architecture to metabolic demands and supply of oxygen and resources (e.g., Atkinson et al., 2006; Czarnoleski, Ejsmont‐Karabin, et al., 2015; Kozłowski et al., 2010). For example, ectotherms often show phenotypic plasticity that results in larger body size under colder rearing conditions (the so‐called temperature–size rule; Atkinson, 1994) or geographic clines that form by evolving larger sizes in colder climates and smaller sizes in warmer climates (the so‐called Bergmann's rule; Bergmann, 1848); evidence shows that these patterns are often associated with changes in cell size (Adrian, Czarnoleski, & Angilletta, 2016; Partridge, Barrie, Fowler, & French, 1994; van Voorhies, 1996; Zwaan, Azevedo, James, van 't Land, & Partridge, 2000). Interestingly, latitudinal clines in the body size of isopods that follow Bergmann's rule have been reported in *Porcellio laevis* woodlice from Chile (Lardies & Bozinovic, 2006), a close relative of *P. scaber*, and it would be interesting to evaluate the contributions of cell size and cell number in these clines.

### 4.3 Oxygen, temperature, cell size, and metabolic performance

In comparing our results with TOCS, we found a partial agreement with predictions that warm and oxygen‐deficient conditions favor small cells (hypothesis 1). As expected, woodlice exposed to chronic oxygen deficiency during development tended (*p* = .07) to have smaller cells consistently in all three studied tissue types (see PC1, Figure 2). Importantly, a decreased cell size under hypoxia could be alternatively viewed as a mechanistic effect of lower macromolecule production caused by a deteriorated supply of ATP. Nevertheless, this scenario is unlikely to explain our results, because it suggests that the hypoxia‐imposed reduction in cell size is inherently linked to reductions in organ and body mass, while in our study, cell size was compared between normoxia and hypoxia in animals of the same body mass. A link between the environmental supply of oxygen and cell size has been very rarely studied, but a decrease in cell size in response to environmental hypoxia was observed, for example, in rotifers (Czarnoleski, Ejsmont‐Karabin, et al., 2015; Walczyńska et al., 2015) and fruit flies (Heinrich et al., 2011; Zhou et al., 2007), but not in bryozoans (Atkinson et al., 2006). Against TOCS predictions, thermal environment did not affect cell size in the studied woodlice (see PC1, Table 2, Figure 2). We cannot determine whether this finding generalizes to other isopods, because to the best of our knowledge, no earlier works have addressed the effects of the thermal environment on isopod cell size. Nevertheless, our findings for *P. scaber* do not follow the results of many previous studies on other invertebrates, which demonstrated that animals originating from warmer or thermally variable environments were characterized by small cells (Adrian et al., 2016; Atkinson et al., 2006; Czarnoleski, Labecka, et al., 2015, but see Arendt, 2006). Certainly, detecting the effects of thermal and/or oxygen conditions during development on cell size can depend on whether the tissue‐specific functions of cells allow for changes in cell shape. If so, even a cell with larger size but a more elongated shape or with plasma membrane microfolds would be able to generate a large plasma membrane area to sustain efficient transmembrane transport, and it is telling that it has been reported that hepatopancreatic cells of isopods change their shape and size in daily secretion cycles (Hames & Hopkin, 1991). However, this perspective on cell size research requires further investigation.

Given the framework of TOCS, we predicted that upon transitioning from cold, normoxic to warm, hypoxic conditions, metabolic performance would be less prone to oxygen limitation in small‐cell versus large‐cell woodlice (hypothesis 2). Our results only partially supported this hypothesis. As outlined above, woodlice reared in two different thermal environments had similar cell size, and consistent with predictions, the metabolic performance of the two groups responded in a similar manner to the transition to metabolically more demanding conditions (decreased oxygen availability combined with increased demand for oxygen). However, against predictions, metabolic performance was comparable in woodlice originating from different oxygen conditions, regardless of cell size differences between hypoxic and normoxic woodlice (see Figures 3 and 4 and the previous paragraph). Surprisingly, our results showed that the transition from cold, normoxic to warm, hypoxic conditions resulted in a more pronounced increase in metabolic rate in large woodlice than in small woodlice, despite the large woodlice being composed of larger cells than small woodlice. We can only speculate on some possible explanations of this discrepancy in our predictions regarding the effects of cell size on metabolic performance, especially given that our data do not allow us to separate the effect of cell size from the effect of body size, as the two traits were strongly interrelated. For example, the properties of the gas‐exchange system of woodlice (e.g., the lungs) might make larger individuals less prone to oxygen limitation than smaller individuals. We should also consider here that cell size and, thus, the total area of plasma membranes in tissue might not affect metabolic performance if animals are studied in their resting state, as was the case here. In contrast to our findings, some previous studies have demonstrated an inverse relationship between cell size and metabolic rate (e.g., Chown et al., 2007; Czarnoleski et al., 2018; Hermaniuk, Rybacki, & Taylor, 2017; Maciak et al., 2014; Starostová et al., 2013; Starostová, Kubička, Konarzewski, Kozłowski, & Kratochvíl, 2009). Also against the pattern revealed here for *P. scaber*, comparisons of thermal sensitivity of Carabidae beetles showed that large species were characterized by higher metabolic responses to thermal change than small species (Gudowska, Schramm, Czarnoleski, Kozłowski, & Bauchinger, 2017). Note, however, that these studies of metabolic rates focused primarily on differences in maintenance costs driven by cell size rather than on the capacity of cells to deliver adequate levels of oxygen under temporal fluctuations in metabolic demand and oxygen supply. In fact, there are arguments against extrapolating physiological phenomena observed in organisms during resting states to ecologically and evolutionarily relevant situations because resting metabolism can be a by‐product of evolution of other characters (Clarke & Pörtner, 2010; Kozłowski et al., 2020).

### 4.4 Residual cell size variance and ommatidia number

By focusing on a relatively small part of the cell size differences among woodlice captured by PC2 in this study (Figure 3, Table 2), we found a very complex pattern: The size of hepatopancreatic cells was negatively related to the size of ommatidia, and these two types of cells changed their size independently of the size of epithelial cells in the hindgut. This pattern resembles earlier reports that cells in anabolically active organs involved in service functions, such as the liver in vertebrates (Czarnoleski et al., 2017, 2018) or the hepatopancreas in invertebrates (Czarnoleski, Labecka, et al., 2015), can undergo changes that are opposite to those of other cell types. Consequently, our analysis of PC2 with reference to predictions that warm hypoxic conditions favor smaller cells (hypothesis 1) leads to inconclusive results. While the size of cells in two tissues (ommatidia and hepatopancreas) changed according to oxygen and thermal conditions during development, the size of cells in the hindgut epithelium was largely irresponsive to developmental conditions. Furthermore, when the size of one cell type decreased in response to high temperature, as predicted by hypothesis 1, the other cell type increased in size, contrary to hypothesis 1. However, this effect of temperature further depended on rearing oxygen level: In hypoxia, there were no differences among cells under different thermal conditions, but in normoxia, cold temperature caused shrinkage of ommatidia and enlargement of hepatopancreatic cells. Despite the complexity of this pattern, the results of PC2 provide some insights into potential sex differences in cell size. We observed that females were characterized by higher PC2 scores than males, suggesting larger cells in the female hepatopancreas and smaller ommatidia in the female compound eye compared to males. According to Kubrakiewicz (1994) and Tseng, Chen, Kou, Lo, and Kuo (2001), the hepatopancreas of crustacean females is involved in vitellogenin synthesis, but the hepatopancreas of isopod females serves only as the storage site for vitellogenin synthesized in fat bodies (Picaud, 1980; Vafopoulou & Steel, 1995). It is tempting to consider this characteristic of isopods in understanding sex differences in hepatopancreatic cell size of *P. scaber*: larger hepatopancreatic cells in females may reflect the amount of vitellogenin storage, which is required by yolk production for oocytes. Note that our results of PC2 combined with the data on the number of ommatidia indicate that *P. scaber* males were characterized by larger but fewer ommatidia than females (Figure 5). Interestingly, no intersexual differences in ommatidia size were reported in the solitary mason bees, *Osmia rufa* (Kierat, Szentgyörgyi, Czarnoleski, & Woyciechowski, 2016), although males had larger ommatidia than females if both sexes were compared at equal body sizes. Compared with *Ligia oceanica* isopods, in which the number of ommatidia reaches 700 (Edwards, 1969), or with insects that have in excess of 1,000 ommatidia (e.g., *Asterocampa leilia* butterflies, Ziemba & Rutowski, 2000), we found that the eyes of *P. scaber* consisted of very few (20–30) ommatidia (Figures 1 and 5). Therefore, it is possible that one or two new ommatidia added to a small eye of *P. scaber* females might have an incomparably large effect on their visual capacity compared with eyes that contain much larger numbers of ommatidia.

Exploring further differences in the number of ommatidia among our experimental treatments, we observed more numerous ommatidia in animals reared at high temperature and a trend toward an increased number of ommatidia under hypoxia. Given that ommatidia size was not affected by temperature, changes in ommatidia number should be tightly linked to changes in the total size of the eye. Earlier works provided evidence suggesting that the eyes of crustaceans and insects might consist of ommatidia with fixed size, and thus, changes in eye size could be obtained exclusively by the addition of new ommatidia (Minelli et al., 2010). Nevertheless, ommatidia size was shown to vary according to rearing temperature in *D. melanogaster* (Azevedo et al., 2002) and *Osmia rufa* (Kierat et al., 2016) and between animals from different nests in *Formica rufa* ants (Perl & Niven, 2016a); it was also shown to change among regions of the eye, as demonstrated in *Asterocampa leilia* butterflies (Ziemba & Rutowski, 2000) and *F. rufa* ants (Perl & Niven, 2016b). Note that our results for *P. scaber* (PC1) also revealed changes in the size of ommatidia with body size and, to a lesser degree, with oxygen level in the environment (Tables 2 and 3). Interestingly, we have good reason to expect that the size of ommatidia can vary according to the region of the eye in woodlice. While imaging, measuring, and counting ommatidia in *P. scaber*, we observed that the smallest and most “packed” ommatidia were present in the frontal part of the eye (unfortunately, we did not measure these ommatidia for technical reasons). We suppose that upon consecutive molting events, new ommatidia are added to the frontal edge of the eye. This phenomenon was already observed in scanning electron microscopy (SEM) images of mancae developmental stages in closely related isopod species, such as *Porcellio dilatatus* (Brum & Araujo, 2007; figures: 46, 47) and *Ligia exotica* (in crescentic dorso‐anterio‐ventral edge of eye; Keskinen, Meyer‐Rochow, & Hariyama, 2002).

## 5 CONCLUSIONS

Overall, we conclude that factors shaping cell size and metabolic performance in *P. scaber* appear more complex than predicted by TOCS and cannot be fully attributed to the effects of thermal and oxygen conditions in the environment. We observed a rather weak influence of rearing conditions on cell size: A tendency toward smaller cells under hypoxia was revealed in the major component of cell size differences among woodlice (our PC1, Figure 2) and a very complex cell size pattern was revealed by the smaller component of cell size differences among woodlice (our PC2, Figure 3). Our evidence also suggests that an organism‐wide coordination of cell growth (PC1) may represent an important mechanistic component of body mass growth in *P. scaber*. It would be interesting to further explore this phenomenon, especially considering that many isopods, including *P. scaber*, evolved life strategies characterized by a continuous allocation to somatic growth after maturation (Antoł & Czarnoleski, 2018). We also revealed two intriguing patterns: a sexual dimorphism of the eyes, with female woodlice tending to have more ommatidia than male woodlice, and an increased metabolic capacity of larger woodlice that consisted of larger cells in a body. To better understand the links among body size, cell size, and metabolic performance, future studies should evaluate metabolic performance at different activity levels and consider the transport efficiencies of different elements of the oxygen delivery system.

## CONFLICTS OF INTEREST

The authors declare no conflicting interests.

## AUTHOR CONTRIBUTION

**Andrzej Antoł:** Conceptualization (supporting); Data curation (lead); Formal analysis (lead); Investigation (equal); Methodology (equal); Software (lead); Validation (equal); Visualization (lead); Writing‐original draft (lead); Writing‐review & editing (lead). **Anna Maria Labecka:** Conceptualization (equal); Investigation (equal); Methodology (lead); Supervision (equal); Writing‐review & editing (equal). **Terézia Horváthová:** Investigation (equal); Writing‐review & editing (supporting). **Anna Sikorska:** Investigation (supporting); Project administration (supporting). **Natalia Szabla:** Investigation (supporting); Writing‐review & editing (equal). **Ulf Bauchinger:** Investigation (supporting); Methodology (equal); Validation (supporting); Writing‐review & editing (supporting). **Jan Kozłowski:** Conceptualization (lead); Funding acquisition (lead); Project administration (lead); Validation (supporting); Writing‐review & editing (supporting). **Marcin Czarnoleski:** Conceptualization (lead); Formal analysis (supporting); Funding acquisition (equal); Methodology (lead); Project administration (supporting); Supervision (lead); Validation (equal); Writing‐review & editing (lead).

## Data Availability

All data used in this manuscript are available at the following link: https://figshare.com/articles/Woodlice_cell_size_data/11604063.
